# Identification of MrtAB, an ABC Transporter Specifically Required for *Yersinia pseudotuberculosis* to Colonize the Mesenteric Lymph Nodes

**DOI:** 10.1371/journal.ppat.1002828

**Published:** 2012-08-02

**Authors:** Gregory T. Crimmins, Sina Mohammadi, Erin R. Green, Molly A. Bergman, Ralph R. Isberg, Joan Mecsas

**Affiliations:** 1 Department of Molecular Biology and Microbiology, Tufts University School of Medicine, Boston, Massachusetts, United States of America; 2 Molecular Microbiology Program, Sackler School of Graduate Biomedical Sciences, Tufts University School of Medicine, Boston, Massachusetts, United States of America; 3 Howard Hughes Medical Institute, Tufts University School of Medicine, Boston, Massachusetts, United States of America; Stanford University School of Medicine, United States of America

## Abstract

A highly conserved virulence plasmid encoding a type III secretion system is shared by the three *Yersinia* species most pathogenic for mammals. Although factors encoded on this plasmid enhance the ability of *Yersinia* to thrive in their mammalian hosts, the loss of this virulence plasmid does not eliminate growth or survival in host organs. Most notably, yields of viable plasmid-deficient *Yersinia pseudotuberculosis* (*Yptb*) are indistinguishable from wild-type *Yptb* within mesenteric lymph nodes. To identify chromosomal virulence factors that allow for plasmid-independent survival during systemic infection of mice, we generated transposon insertions in plasmid-deficient *Yptb*, and screened a library having over 20,000 sequence-identified insertions. Among the previously uncharacterized loci, insertions in *mrtAB*, an operon encoding an ABC family transporter, had the most profound phenotype in a plasmid-deficient background. The absence of MrtAB, however, had no effect on growth in the liver and spleen of a wild type strain having an intact virulence plasmid, but caused a severe defect in colonization of the mesenteric lymph nodes. Although this result is consistent with lack of expression of the type III secretion system by Wt *Yptb* in the mesenteric lymph nodes, a reporter for YopE indicated that expression of the system was robust. We demonstrate that the ATPase activity of MrtB is required for growth in mice, indicating that transport activity is required for virulence. Indeed, MrtAB appears to function as an efflux pump, as the ATPase activity enhances resistance to ethidium bromide while increasing sensitivity to pyocyanin, consistent with export across the inner membrane.

## Introduction

There are three mammalian pathogens in the *Yersinia* genus: *Yersinia pseudotuberculosis*, *Yersinia pestis*, and *Yersinia enterocolitica*. *Yersinia pesti*s, the causative agent of plague, has had profound effects on human civilization, killing one out of three people in Europe during one epidemic, the Black Death [1]. In contrast, the highly related pathogens *Y. pseudotuberculosis* (*Yptb*) and *Y. enterocolitic*a (Ye) usually cause self-limiting gastrointestinal infections. Although they differ in the route of infection and disease outcome, all three *Yersinia* species share a tropism for growth in lymph nodes. Infection with *Y. pestis* often results in dramatically inflamed lymph nodes, while *Yptb* or *Ye* infections are associated with acute mesenteric lymphadenitis due to their tropism for colonization of the mesenteric lymph nodes [2]. All three pathogenic *Yersinia* species also share a conserved virulence plasmid, which encodes a Type III Secretion System and its associated translocated substrate proteins, called Yops [3].

The virulence plasmid is required for optimal *Yptb* growth in a variety of mammalian organs, including the small intestine, cecum, Peyer's patches, liver, spleen, and lung [4], [5]. Detailed study of the components of the virulence plasmid, including the TTSS, Yops, and the adhesin YadA, has revealed that each are required during growth in these same organs [6], [7], [8]. It is believed that the Yops in particular are required to disarm many components of the host innate immune response, with hypothesized functions including interfering with phagocytosis and misregulating immune signaling pathways [6], [9]. Given this background, it was surprising when it was revealed that the loss of the virulence plasmid and its arsenal of encoded Yops did not reduce the growth of *Yptb* in the mesenteric lymph node, as measured by colony forming units [5]. The mesenteric lymph node, therefore, behaves anomalously in that the chromosomally encoded *Yptb* virulence factors appear to be sufficient for growth in the MLN.

Several genetic screens have been performed in pathogenic *Yersinia* species in pursuit of virulence factors required during animal infection [10], [11], [12], [13]. There are a number of chromosomally-encoded factors required for efficient systemic disease or during intestinal colonization, including invasin, PhoP/PhoQ, Ybt, and pH 6 antigen [14], [15], [16], [17]. However, the previous genetic screens were limited in the number of genes that were analyzed. In addition, no systematic identification of proteins encoded by the chromosome, in the absence of contributions from the virulence plasmid, has been performed. Therefore, we hypothesized that there are multiple chromosomal *Yptb* virulence factors yet to be discovered.

In this study, we describe the screening of over 20,000 plasmid-deficient sequence-identified transposon insertion mutants in mice, and the identification of a number of candidate virulence factors. We identified *mrtAB*, encoding a previously uncharacterized heterodimeric ABC transporter that is critical for the growth and persistence of plasmid-cured *Yptb* in mice. Intriguingly, *mrtAB* is only necessary for Wt *Yptb* (P^+^) to colonize a single organ, the mesenteric lymph node.

## Results

### Characterization of *Yersinia pseudotuberculosis* (P^−^) colonization of mouse organs and determination of the bottleneck size

**Figure 1:**
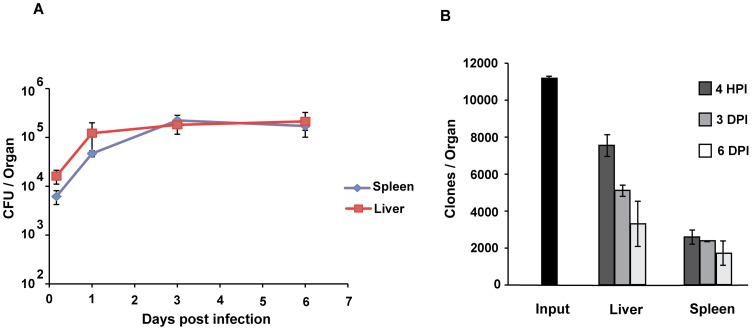
Plasmid deficient *Yersinia pseudotuberculosis* grow and persist in mouse deep tissue sites with little clonal loss. A) Growth within the spleen (blue diamond) and liver (red square) after IV inoculation of *Yptb*(P^−^) in C57BL/6 mice. 1×10^5^ *Yptb*(P^−^) were IV inoculated, organs were collected 3 days post-infection, and bacterial numbers were determined by colony forming units (CFU) per organ. N = 3–6 mice, mean CFU is plotted, ± standard deviation. B) Average number of unique transposon insertion clones in the Input library, and per organ over 6 days post IV infection, +/− standard deviation, N = 3.

As plasmid-deficient *Yersinia pseudotuberculosis* (*Yptb* (P^−^)) persists in various host organs [5], [18], we decided to perform a genetic screen for chromosomal *Yptb* virulence factors. Previous studies indicated that the number of clones that colonize the small intestine or successfully invade internal organs after oral inoculation was small [10], [19]. Therefore, to increase the number of mutants that could be analyzed in a single mouse infection, we investigated the ability of *Yptb* (P^−^) to infect mouse spleens and livers following intravenous (IV) injection. IV injection of 10^5^ *Yptb* (P^−^)revealed that approximately 10% of the inoculum were present in the liver or spleen at 4 hours post-infection (Fig. 1A). Furthermore, the bacteria present in these organs were able to persist for over a week and also exhibited roughly 30-fold growth over this time period (Fig. 1A).

Although we were able to get a rough estimate of the number of clones that colonized the spleen and liver by looking at viable counts of bacteria in these organs at 4 hours post-infection, it was unclear how many of these clones would survive after facing the full onslaught of the host immune system for days. For example, the increase in CFU in the liver from 10^4^ to over 10^5^ between 4 hours and 3 days post-infection could represent the loss of 99% of the clones, followed by 1,000-fold growth of each remaining clone, or it could represent over 10-fold growth of each clone (narrow or wide “bottleneck,” respectively). This is a critical distinction whenever performing a genetic screen in an animal, as it determines how many mutants one can screen per mouse.

To determine the size of the bottleneck for *Yptb*(P^−^), the number of clones in the liver and spleen that initially entered these organs, and whether the clonal number is reduced over time, we used TnSeq, an insertion mutagenesis procedure that allowed us to follow the fate of individual insertions by deep sequencing of the entire pool of insertion sites before and after inoculation [20]. As the protocol that we described resulted in no more than 10^4^ clones establishing residence in the liver or spleen, we generated libraries of approximately 10^4^ mariner transposon mutants, which should represent the maximum number of clones present in each organ (Experimental Procedures). One of the pools of 10^4^ mutants was inoculated into mice; bacteria were isolated from the liver and spleen at 4 hours, 3 days, or 6 days post-infection; and genomic DNA was isolated from the bacterial colonies derived from these organs (Experimental Procedures). We then performed deep sequencing on the insertion sites [20] to identify the number of clones that survived over this time period, and found about 7,600 and 2,600 clones in the liver and spleen, respectively, that initially established residence in these organs (Fig. 1B). While there was loss of clones over time, the persistence of the vast majority of clones over 3 days was striking, given that these plasmid-deficient bacteria lack many of the known virulence factors. By 6 days post-infection the number of clones in the liver was less than half the number that colonized, and the variance increased as in some mice, more clones were lost than in others.

### Identification of *Yptb*(P^−^) mutants that are depleted from deep tissue sites

**Figure 2:**
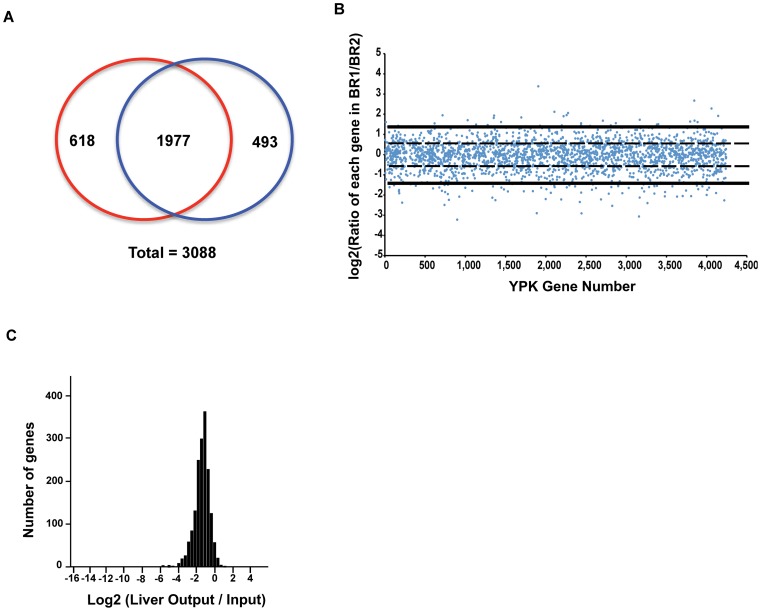
Genetic screen for chromosomal *Yptb* virulence factors. A) Number of genes that were mutated in each input library, and the number of genes mutated in both libraries. Red = library #1, Blue = library #2. B) Quality control of 2 biological replicates (BR) of the Input Library #2, sequenced separately. Gene number is on the X axis (*Yptb* has 4250 genes), and log_2_(ratio of each gene in BR 1/BR 2) is on the Y axis. Dashed line = 1 standard deviation, solid line = 2 standard deviations C) Histogram of 1977 genes mutated in both library #1 and library #2, X axis = log_2_(Average ratio of Liver Output/Input), Y axis = number of genes that have a threshold X value. The X axis extends to include values for all genes.

Based on the clonal analysis (Fig. 1B), we chose to screen for mutants that were defective for growth/persistence in the liver 3 days after IV inoculation to maximize the number of mutants that could be screened and allow growth within these tissue sites. Each library of approximately 10,000 mutants was screened through 10 mice, for a total of over 20,000 independent transposon insertion mutants, encompassing 3,088 genes (Fig. 2A). The “output” samples for the screen were the pooled colony forming units (CFU) from each individual infected liver, and the “input” samples were the pooled CFU from each library culture prior to inoculation. The bacteria were then scraped off the plate, genomic DNA was isolated, and the abundance of each Tn insertion was quantified for each output and input sample using deep sequencing (Experimental Procedures; [20]). Biological replicates of the input samples displayed very little variability (Fig. 2B) confirming the reproducibility of the method. Preparation of the input pool involved growth of the bacteria in culture at 26°C prior to inoculation of mice, so mutants defective for growth in mice could simply be temperature sensitive for growth. To identify insertions that were depleted in the liver and also had general defects in growth at elevated temperatures, we also performed a control screen by preparing a control pool grown at 37°C in culture, and compared this pool to the input grown at 26°C (Table 1 and Table S4).

**Table 1:** Identification of mutants defective for colonization in liver.

Functions	Gene	Library	Annotation	Output/Input	37/26
**Known virulence factors**					
	YPK_2757	#1 and #2	pH 6 Ag	1.07E-02 (−4.72)	1.15 (0.39)
	YPK_0665	#1 and #2	sufI	1.86E-02 (−3.99)	0.38 (−1.95)
	YPK_2429	#1 and #2	invasin	1.98E-02 (−3.91)	1.23 (0.53)
	YPK_2759	#1 and #2	pH 6 Ag	3.08E-02 (−3.32)	0.83 (−0.29)
	YPK_2758	#2	pH 6 Ag	0 (NA)	ND
**AA and Purine Synthesis**					
	YPK_0321	#1 and #2	aroE	3.28E-04 (−9.27)	0.23 (−3.05)
	YPK_1253	#1 and #2	purM	5.28E-03 (−5.67)	0.96 (0.002)
	YPK_0226	#1 and #2	aroB	1.29E-02 (−4.48)	0.28 (−2.62)
	YPK_0357	#1 and #2	purH	1.80E-02 (−4.04)	0.66 (−0.8)
	YPK_2670	#1 and #2	aroA	3.06E-02 (−3.33)	0.44 (−1.65)
	YPK_2047	#1 and #2	trpA	3.33E-02 (−3.22)	0.61 (−1.0)
	YPK_2528	#1 and #2	hisB	3.42E-02 (−3.18)	0.87 (−0.22)
	YPK_0356	#1	purD	9.53E-03 (−2.58)	0.82 (−0.33)
	YPK_1364	#2	purC	0 (NA)	0.57 (1.1)
**LPS modification**					
	YPK_3181	#1 and #2	O-Ag	0 (NA)	0.22 (−3.17)
	YPK_3646	#1 and #2	waaL	0 (NA)	0.02 (−8.55)
	YPK_4033	#1 and #2	wecA	0 (NA)	0.0008 (−15.03)
	YPK_3937	#1 and #2	rfaH	2.36E-05 (−12.87)	0.52 (−1.31)
	YPK_3184	#1 and #2	O-Ag	1.52E-03 (−7.32)	0.61 (−0.95)
	YPK_3190	#1 and #2	O-Ag	1.98E-03 (−6.97)	0.24 (−2.91)
	YPK_3179	#1 and #2	O-Ag	2.32E-03 (−6.77)	1.09 (0.27)
	YPK_3183	#1 and #2	O-Ag	5.38E-03 (−5.64)	0.37 (−2.02)
	YPK_3182	#1 and #2	O-Ag	8.05E-03 (−5.11)	0.23 (−3.06)
	YPK_3189	#1 and #2	O-Ag	2.05E-02 (−3.87)	0.15 (−4.0)
	YPK_4030	#1 and #2	wecC	2.61E-02 (−3.55)	1.03 (0.16)
	YPK_1834	#1 and #2	**arnD**	4.77E-02 (−2.74)	0.69 (−0.71)
	YPK_3180	#1	O-Ag	8.83E-03 (−2.64)	0.16 (−3.74)
	YPK_3188	#2	O-Ag	2.63E-04 (−5.42)	0.25 (−2.86)
**Candidate virulence factors**					
	YPK_3221	#1 and #2	mrtB	0 (NA)	0.73 (−0.58)
	YPK_3222	#1 and #2	mrtA	2.19E-03 (−6.84)	0.77 (−0.46)
	YPK_1234	#1 and #2	phage protein	2.69E-03 (−6.57)	0.68 (−0.73)
	YPK_2423	#1 and #2	flgD	3.03E-02 (−3.34)	1.03 (0.16)
	YPK_1292	#1 and #2	rodZ	4.07E-02 (−2.95)	0.48 (−1.45)
	YPK_2066	#1 and #2	oppD	4.40E-02 (−2.85)	1.05 (0.19)
	YPK_3575	#1 and #2	apaH	4.48E-02 (−2.82)	0.18 (−3.6)
	YPK_1713	#1 and #2	Hypothetical	5.20E-02 (−2.63)	1.05 (0.2)
	YPK_2406	#1	Hypothetical	0 (NA)	1.75 (1.28)
	YPK_3656	#1	Hypothetical	0 (NA)	0.78 (−0.45)
	YPK_0453	#1	tRNA synthase	1.73E-04 (−5.98)	1.59 (1.08)
	YPK_0688	#1	Hypothetical	4.46E-04 (−5.17)	ND
	YPK_2424	#1	flgC	8.56E-03 (−2.67)	2.26 (1.83)
	YPK_3600	#1	Hypothetical	9.96E-03 (−2.54)	ND
	YPK_2199	#2	Hypothetical	0 (NA)	0.85 (−0.27)
	YPK_4078	#2	sthA	6.57E-03 (−2.96)	0.3 (−2.49)
	YPK_0208	#2	Hypothetical	8.67E-03 (−2.75)	0.85 (−0.26)

Shown are gene insertions that were ≥2.5 s.d. depleted from the pool relative to the mean, based on number of sequencing reads (Experimental Procedures). Library: library harboring the mutations in noted gene; Output/Input: average ratio of the relative abundance of clones containing transposon insertions in gene in the Output liver sample/Input liver sample (± sd); 37/26: average ratio of the relative abundance of clones containing transposon insertions in gene after growth in broth at 37°/growth in broth at 26°C (± s.d.). Data are separated into categories of known virulence factors, amino acid and purine synthesis, LPS modification, and novel candidate virulence factors.

All the data for insertions in a given gene were then analyzed, and average ratios for output/input was determined for each gene, using colonies isolated from each liver as a separate output and colonies isolated from the injection dose as input (Fig. 2C). Genes of interest were identified as having a log2 normalized output/input ratio of ≥2.5 s.d. from the mean. The data from the screen are summarized in Table 1.

Insertion mutations that fulfilled the above criteria were identified in 4 main categories of genes (Table 1): 1) Genes encoding proteins previously shown to be required for disease in animal models (Known Virulence Factors; [10], [17], [21]; 2) Genes encoding proteins predicted to be involved in amino acid or nucleic acid synthesis; 3) Genes encoding proteins known to be involved in LPS modification, especially O-antigen (O-Ag) synthesis; and 4) Uncharacterized genes or other genes encoding proteins not previously known to be important in previous *Yersinia* models of disease (candidate virulence factors). Identification of 5 genes known to encode proteins implicated in virulence, including the genes for pH 6 antigen (mutations in 3 genes), invasin, and SufI [10], provided excellent positive controls for the screen, and was consistent with the screen being able to identify proteins that are important in a *Yptb*(P^+^) background (Table 1). Mutations in genes encoding proteins involved in amino acid and purine synthesis have been previously identified in screens for mutants defective for disease in animal models, and several orthologs of the genes identified in Table 1 are also required for disease in related pathogens such as *Salmonella enterica* serovar Typhimurium [22]. The 14 genes required for LPS modification, particularly in regards to O-Ag, fell into 2 categories as well: those that are required for growth at elevated temperatures, and those that are not. For example, the genes that encode for the predicted O-Ag ligase (YPK_3646) and WecA (YPK_4033) are both required for growth at 37°C. In contrast, a number of the genes predicted to be involved in LPS and O-Ag modification and synthesis are not required for growth at 37°C, and several have an intermediate, minor defect at 37°C (Table 1).

### Identification and characterization of novel *Yptb* virulence factor, *mrtAB*

**Figure 3:**
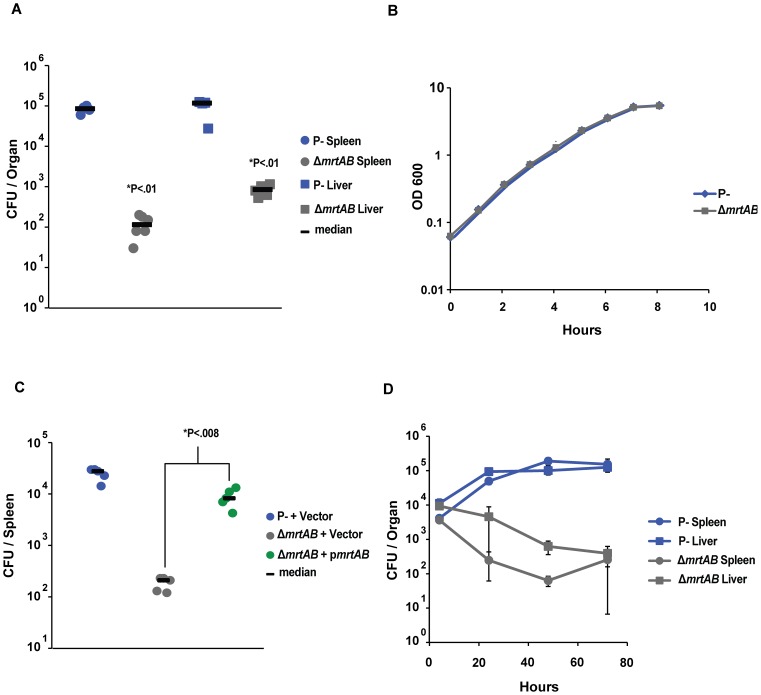
*mrtAB* is required for growth of *Yptb* (P^−^) in liver and spleen. **A**) In frame deletion of *mrtAB* in *Yptb*(P−) recapitulates the data from the screen. Mice were inoculated IV with 1×10^5^ bacteria, organs were collected 3 days post-infection, and bacterial number was determined by colony forming units (CFU) per organ. N = 4–6 mice. **B**) Deletion of *mrtAB* does not alter growth at 37° in 2XYT broth culture. Data are mean of 3 replicates, error bars = ± standard deviation. **C**) Rescue of *Yptb*(P^−^) *ΔmrtAB* in trans with p*mrtAB*. Mice were injected IV with 1×10^5^ *Yptb* (P^−^)/Vector, *Yptb* (P^−^)Δ*mrtAB*/Vector, or *Yptb* (P^−^) Δ*mrtAB/*p*mrtAB*, spleens were collected 3 days post-infection and analyzed as in A. N = 5 mice. **D**) Growth curve of *Yptb* (P^−^) and *Yptb* (P^−^) Δ*mrtAB* in liver and spleen over 3 days. Mice were injected IV with 1×10^5^ *Yptb* (P^−^) and *Yptb* (P^−^) Δ*mrtAB*, organs were collected between 4 hours and 3 days post-infection, and analyzed as in A. N = 3 mice, +/− standard deviation. *Statistical significance (P*) in Figure 3 was determined by nonparametric Mann–Whitney test.

Of the mutations in the previously uncharacterized genes, insertions in two contiguous genes, YPK_3222-3221 encoding a predicted heterodimeric ABC transporter, had the most severe defects. To verify that the defect predicted by the TnSeq analysis can be repeated during mouse infections using single strains, an in-frame deletion removing both genes in the plasmid-cured *Yptb*(P^−^) was generated. Three days after IV inoculation, this strain gave yields between 10^2^ and 10^3^ lower than the parental strain in the liver and spleen, respectively (Fig. 3A). Similar results were obtained with individual deletions of these genes as expected for a heterodimeric transporter (data not shown). The lowered presence of this mutant in deep tissue sites after IV inoculation of mice was not due to a general growth defect or temperature sensitive growth, as the knockout removing both genes grew identically to the parental strain in broth culture at 37°C (Fig. 3B). The splenic colonization phenotype of the YPK_3222-3221 deletion mutant was almost completely complemented when the two genes were placed on a low copy number plasmid in *trans*, as the yields in deep sites of the deletion mutant containing the complementing plasmid were similar to those of WT harboring an empty vector as control (Fig. 3C). The putative ABC transporter deletion mutant did not exhibit a failure to survive transit through the blood or an inability to colonize the spleen or liver, as the number of bacteria in these organs 4 hours after IV inoculation was identical to Wt *Yptb* (P−) (Fig. 3D). These experiments demonstrated that insertion mutations in the ABC transporter genes resulted in defective growth or persistence in deep tissue sites and did not cause an initial colonization defect or defective growth in culture.

**Figure 4:**
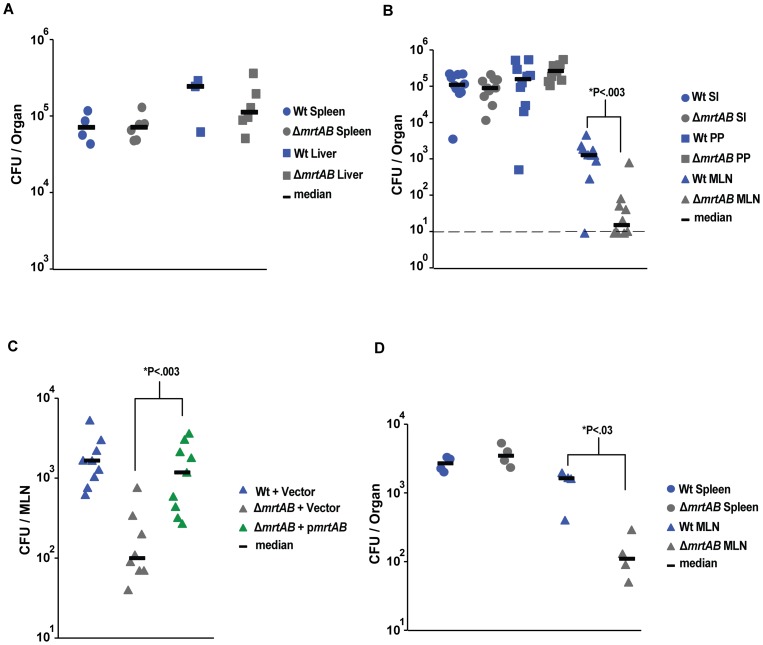
In Wt *Yptb*, *mrtAB* is only required in the mesenteric lymph node. **A**) Growth of *Yptb* (P^+^) in spleen and liver is unaffected by the absence of *mrtAB*. Mice were inoculated IV with 10^3^ *Yptb* (P^+^) derivatives, organs were collected 3 days post-infection, and bacterial number was determined by colony forming units (CFU) per organ. N = 4 or 6. **B**) *Yptb* (P^+^) requires MrtAB for optimal colonization of mesenteric lymph nodes. Mice were orally inoculated with 2×10^9^ *Yptb*(P^+^) or *Yptb*(P^+^)Δ*mrtAB*, organs were collected at 1 day post-infection, and analyzed as in A. The dashed line indicates the limit of detection. N = 10 mice. **C**) Defect in MLN colonization caused by absence of MrtAB is rescued in trans by intact *mrtAB*. Mice were orally inoculated with 2×10^9^ *Yptb*(P^+^)/vector, *Yptb*(P^+^)Δ*mrtAB*/vector, or *Yptb*(P^+^)Δ*mrtAB/*p*mrtAB*, organs were collected 1 day post-infection and analyzed as in A. N = 8 or 9 mice. **D**) The defect in MLN colonization caused by the absence of MrtAB can be recapitulated after intraperitoneal inoculation. IP inoculation was performed with 2×10^5^ *Yptb*(P^+^)or *Yptb*(P^+^)Δ*mrtAB Yptb*. Organs were collected 1 day post-infection, and analyzed as in A. N = 4 mice. *Statistical significance was determined by nonparametric Mann–Whitney test.

Next we set out to determine the phenotype of the YPK_3222-3221 deletion mutant in a *Yptb* (P^+^) background. Surprisingly, even though the absence of the predicted ABC transporter lowered yields of the P^−^ strain in the liver and spleen, there was no apparent defect in these organ sites after IV inoculation when the same mutation was made in the *Yptb*(P^+^) background (Fig. 4A). Instead, deletion of YPK_3222-3221 in *Yptb* (P^+^) resulted in a defect in only one organ, the mesenteric lymph nodes (MLN). Oral infection of mice with WT or the deletion mutant revealed that bacteria lacking the putative ABC transporter were fully capable of persisting in the small intestine and exponentially increasing their numbers in the Peyers patches as rapidly as Wt, but had almost a 100-fold defect in the colonization or early growth in the MLN (Fig. 4B). We were able to rescue this defect *in trans* using a plasmid harboring both genes, demonstrating that the predicted ABC transporter genes are essential for early colonization of the MLN (Fig. 4C). In addition, this early defect in the MLN was independent of route of administration, as the deletion mutant had over a 10 fold defect in the MLN following intraperitoneal infection, while the colonization of the spleen was equal to WT (Fig. 4D). As the WT strains show a specific defect for MLN colonization, we propose that this operon (YPK_3222-3221) be named *mrtAB*, for Mesenteric lymph node Required Transporter.

### The predicted ATP binding site of MrtB is required for survival in vivo

**Figure 5:**
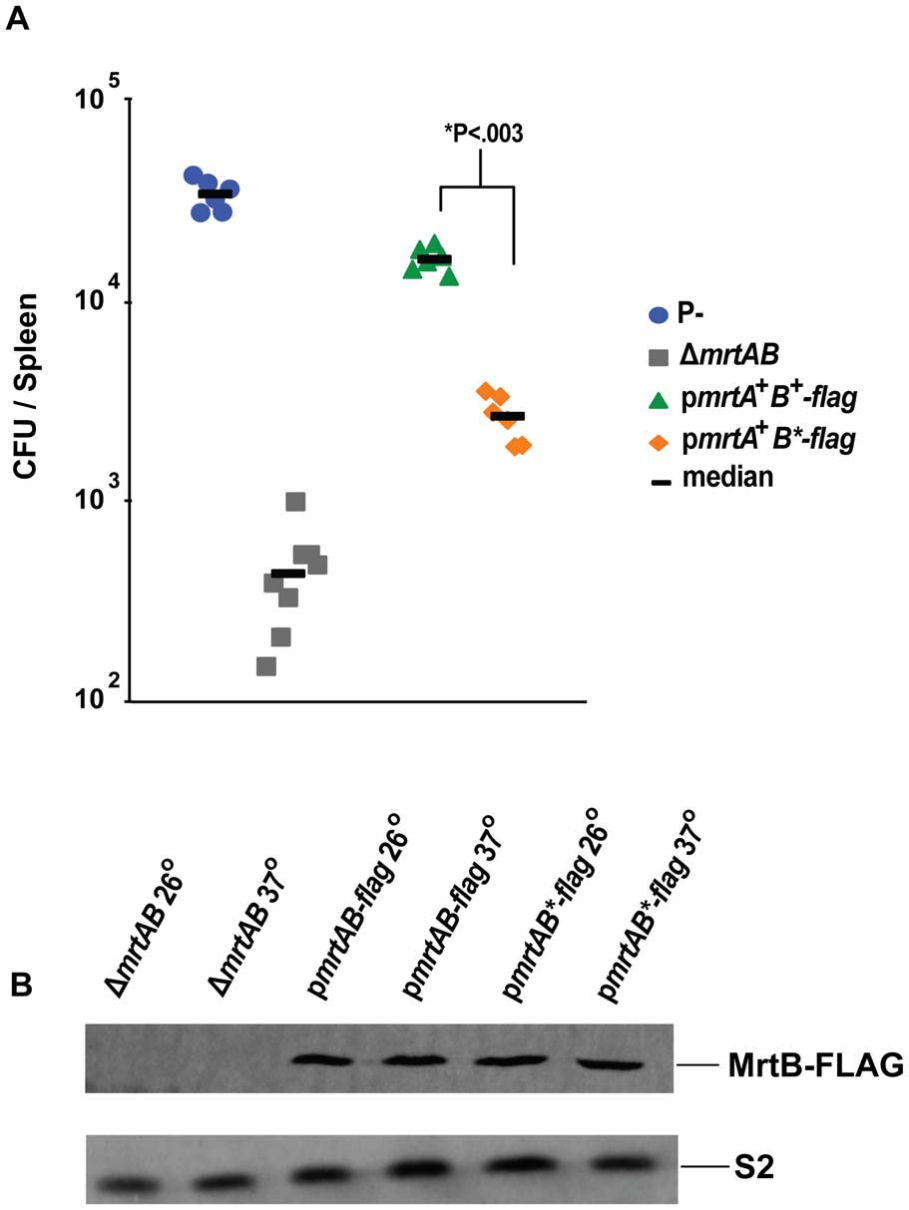
ATPase activity of MrtB is required for optimal growth in vivo. **A**) Mice were infected IV with 1×10^5^ *Yptb*(P^−^)/vector, *Yptb*(P^−^)Δ*mrtAB*/vector, *Yptb*(P^−^)Δ*mrtAB*/p*mrtA* ^+^ *mrtB* ^+^-*flag* complementation vector, or *Yptb*(P^−^)Δ*mrtAB*/p*mrtA* ^+^ mrtB*-flag complementation vector with *K380A mutation in MrtB. Spleens were collected 3 days post infection, and bacterial number was determined by colony forming units (CFU) per organ. N = 6 or 8 mice. **B**) Bacteria were grown *in vitro* to examine the effect of disrupting the MrtB-FLAG Walker A box on MrtB-FLAG expression. *Yptb*(P^−^) Δ*mrtAB*/vector (Lane 1 = 26°C, Lane 2 = 37°C), *Yptb*(P^−^)Δ*mrtAB*/p*mrtA* ^+^ *mrtB* ^+^ *-flag* (Lane 3 = 26°C, Lane 4 = 37°C), or *Yptb*(P^−^)Δ*mrtAB*/p*mrtA* ^+^ mrtB*-flag (*K380A) (Lane 6 = 26°C, Lane 7 = 37°C), were grown in LB *in vitro* at 26° or 37°. Blots were stripped and re-probed with S2 antibody for a loading control. *P: Statistical significance was determined by nonparametric Mann–Whitney test.

The ATPase activity of ABC family transporters is the driving force behind either the export or import of cargo across the membrane [23]. To determine if the nucleotide binding sites of MrtB was necessary for growth of *Yptb* in vivo, we made a point mutation, predicted to disrupt ATP binding in the MrtB Walker A box, on the p*mrtAB* complementation plasmid. The analogous amino acid change has been used to disrupt the ATPase activity of other ABC transporters [24], [25]. We also tagged MrtB with a FLAG tag to allow us to determine if the ATPase mutation reduced steady state levels of the protein. We then tested the ability of this mutated gene to rescue the growth of *Yptb*(P^−^)*ΔmrtAB* in the spleen (Fig. 5). The FLAG-tagged MrtB on the plasmid encoding *mrtAB* (p*mrtA^+^B^+^-flag*) rescued growth of the Δ*mrtAB* strain in the spleen to the same extent as a wild type version of the gene (Fig. 3C, 5A). Disruption of MrtB Walker A box in the *mrtAB*-*flag* complementation construct (pmrtA^+^B*-flag) resulted in a six-fold decrease in yield in the spleen 3 days post-inoculation (Fig. 5A), without noticeably affecting protein expression (Fig. 5B). These experiments indicate that the ATPase activity of MrtB is required for growth of *Yptb*(P^−^) in vivo. We attempted to perform an analogous experiment with MrtA, except we placed a peptide tag (HA) at the N terminus of the protein in an effort to avoid disruption of the *mrtAB* operon, where the 3′ end of the *mrtA* coding region overlaps with the 5′ end of the *mrtB* gene. While we observed that mutation of the MrtA Walker A box had no negative effect on the rescue of *Yptb* (P^−^)*ΔmrtAB* by HA-mrtA*mrtB^+^ in the spleen (data not shown), we were also unable to detect either the HA-tagged MrtA or the mutated HA-tagged MrtA proteins, possibly due to cleavage of HA along with the signal sequence, complicating any interpretation of this result.

### Wt *Yptb* express YopE, and are associated with neutrophils, in the MLN

Based on the results detailed above, it appeared that the virulence plasmid bypassed a requirement for MrtAB in all organs except the MLN. It is known that after oral inoculation, yields of *Yptb*(P^−^) in the MLN are indistinguishable from a virulence plasmid-containing strain even though *Yptb*(P^−^) exhibits a growth defect in every other organ tested [5]. One hypothesis that could explain these observations is that in the MLN, a large proportion of *Yptb* do not express the plasmid-encoded TTSS and Yops, making MrtAB essential for growth in this organ site. A second hypothesis is that the bacteria interact with different sets of innate immune cells in the MLN and the spleen, creating two distinct selective environments for *Yptb* in these organ sites.

**Figure 6:**
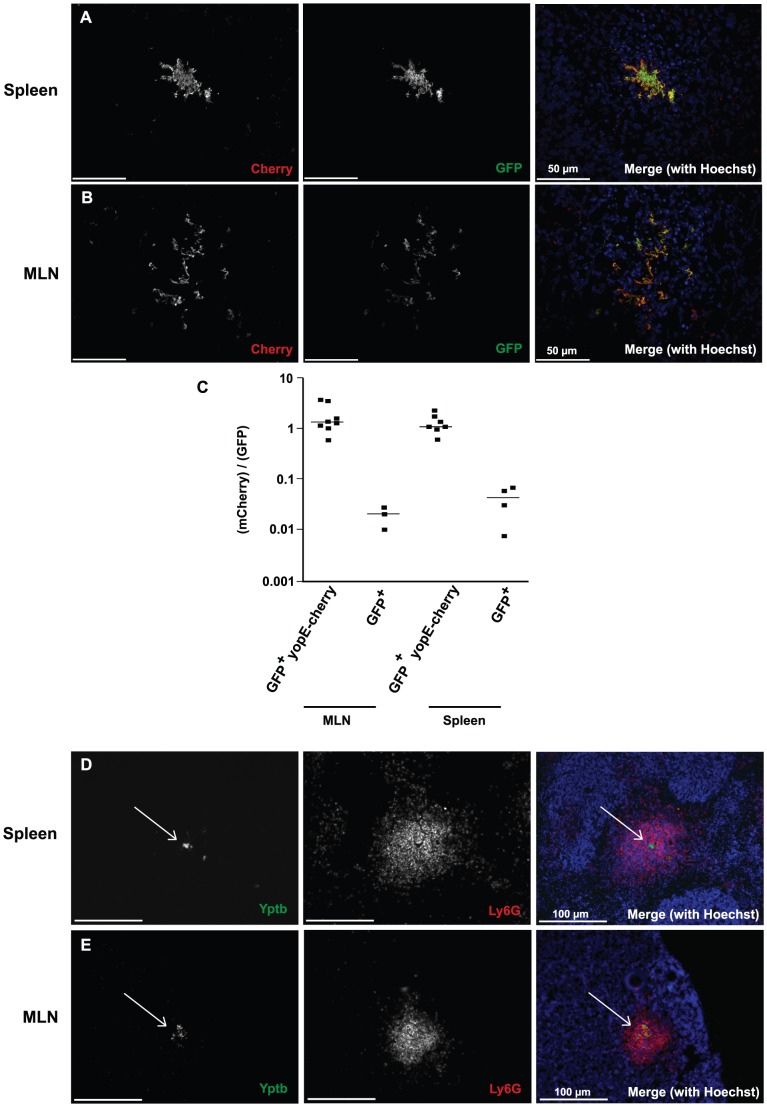
MLN-localized *Yptb*(P^+^) expresses YopE and is in contact with neutrophils. Mice were orally inoculated with 2×10^9^ (MLN) or injected IV with 10^3^ (Spleen) bacteria to approximately synchronize the infections, organs were collected 2 days post-inoculation, and tissue sections were stained for DNA (Hoechst). Displayed are representative images of *Yptb*(P^+^)-GFP/YopE-mCherry micro-colonies in the spleen (**A**) or MLN (**B**). (Median mCherry fluorescence intensity)/(median GFP fluorescence intensity) for *Yptb*(P^+^)/GFP/YopE-mCherry micro-colonies, or *Yptb*(P^+^)/GFP micro-colonies in the spleens and MLNs, is graphed in **C**. Each symbol in C represents a different focus of infection. N = 3 (GFP control) or N = 7–8 (Reporter). **D**) Spleens from mice infected IV with 1×10^3^ GFP-Wt *Yptb*, or **E**) MLN from mice orally inoculated with 2×10^9^ GFP-Wt *Yptb*, were isolated 2 days post-infection, and tissue sections were stained for neutrophils (Ly6G) and DNA (Hoechst). D and E are representative images.

To test the first hypothesis, we constructed a reporter in which the gene for the fluorescent mCherry protein is transcriptionally fused downstream from an intact *yopE* on the virulence plasmid. The *yopE-mCherry* construction was regulated in the same fashion *as yopE* during growth in broth culture, as bacteria encoding the fusion displayed thermally-induced mCherry expression that required the transcription factor LcrF [26] (Fig S1). While the mCherry protein was stable (data not shown), when bacteria were moved from inducing to non-inducing culture conditions, its expression levels were accordingly diluted (Fig S1). This made the fusion a useful tool for analyzing YopE expression at the single cell level, in a replicating pool of bacteria that were detectable by constitutive GFP expression. We aimed to compare the expression of the YopE reporter in spleens and MLN; however, during oral infection the spleen was shown to be colonized much later than the MLN [5]. Therefore, to approximately synchronize the WT-*yopE^+^*mCherry infections, we compared spleens from mice infected 2 days post IV inoculation, to MLN from mice 2 days post oral inoculation. Contrary to our hypothesis, we found that the expression of the YopE reporter was identical in the spleen and MLN (Fig. 6A–C).

It was recently shown that the TTSS secreted Yops are preferentially found inside neutrophils in the Peyer's patches, MLN, and spleen, at 5 days post-infection, indicating an intimate interaction between *Yptb* and neutrophils during a late stage of the infection [27]. We hypothesized that at earlier stages post-inoculation, when there is a large difference in the requirement of MrtAB in the spleen vs. MLN, there would be altered co-localization of *Yptb* with neutrophils in these organ sites. Instead, we found at 2 days post-infection that the *Yptb* bacterial foci in the spleen and MLN displayed similar levels of co-localization with neutrophils, with 7/8 foci in the MLN, and 28/28 foci in the spleen strongly co-localizing with neutrophils (Fig. 6D–E). The bacterial colonies in the MLN did appear to have a more diffuse morphology than the colonies in the spleen, but still equally co-localized with neutrophils. Therefore, it appears that *Yptb* colonies in the spleen and MLN must all contend with this potent innate immune cell.

### *mrtAB* deficiency results in delayed growth in the MLN, but normal spleen colonization and lethality during oral infection of mice

**Figure 7:**
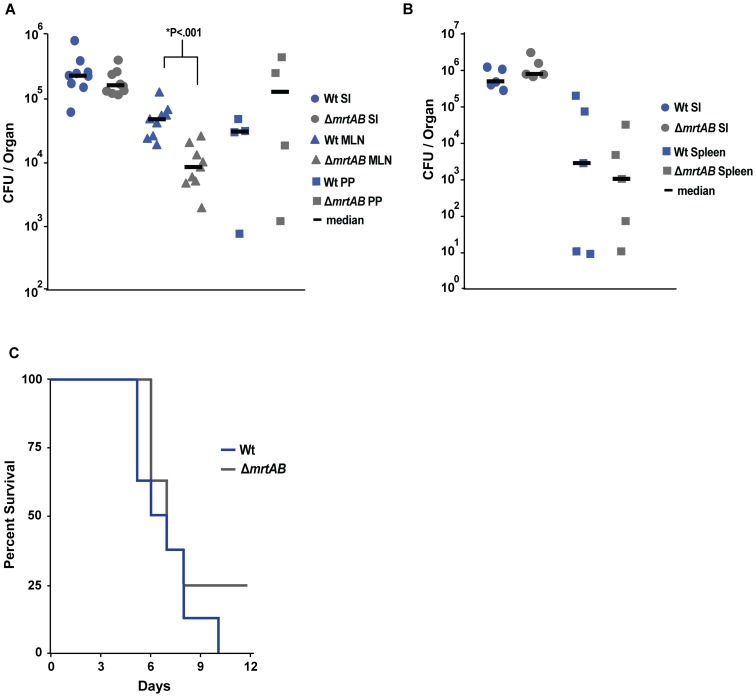
The *Yptb*(P+) *mrtAB* mutant exhibits delayed growth in the MLN, but equal spleen colonization and lethality in mice. **A**) MrtAB deficient *Yptb*(P+) exhibit a modest decrease on MLN colonization at 4 days post-infection. Mice were orally inoculated with 2×10^9^ *Yptb*(P^+^) or *Yptb*(P^+^)Δ*mrtAB*, and the small intestines, MLNs, and PPs were collected at 4 days post-infection, and bacterial number was determined by colony forming units (CFU) per organ. N = 4 mice (PP) or 9 mice (SI and MLN). *P: Statistical significance was determined by nonparametric Mann–Whitney test. **B**) *Yptb*(P+) and *Yptb*(P^+^)Δ*mrtAB* display equal early colonization of the spleen following oral infection. Mice were orally inoculated with 2×10^9^ *Yptb*(P^+^) or *Yptb*(P^+^)Δ*mrtAB*, and the small intestines and spleens were collected at 2 days post-infection, and analyzed as in A. N = 5 mice. **C**) *Yptb*(P+) and *Yptb*(P^+^)Δ*mrtAB* are equally lethal following oral infection with 10^9^ bacteria. Mice were orally inoculated with 10^9^ *Yptb*(P^+^) or *Yptb*(P^+^)Δ*mrtAB*, and the survival of infected mice was monitored over 12 days. N = 8 mice.

To shed light on the role of MrtAB during a later stage of oral infection, we compared the bacterial burden of Wt *Yptb*(P+) and the *mrtAB* mutant in the small intestine, PP, and MLN 4 days post-infection. MrtAB was not required for *Yptb*(P+) to persist in the small intestine or the PP following oral infection (Fig. 7A). Interestingly, while there were roughly 5-fold less Δ*mrtAB Yptb*(P+) in the MLN compared to Wt, the mutant had largely caught up to the Wt *Yptb*(P+) numbers in this organ by 4 days post-infection (compared to 100 fold less of the mutant at 1 day post infection), suggesting that the primary role of MrtAB may be during the initial colonization of the MLN rather than growth after the bacteria establish a replication site in this organ (Fig. 4B, 7A). To determine if MrtAB was generally required for colonization of multiple organs following oral infection, we tested the ability of the *mrtAB* mutant to colonize the spleen 2 days post-infection. Interestingly, MrtAB appeared to be specifically required for MLN colonization, as the mutant colonized the spleen at a level equal to Wt *Yptb*(P+) (Fig. 7B). Consistent with the similar ability of Wt and MrtAB deficient *Yptb*(P+) to colonize internal organs such as the spleen, Wt *Yptb*(P+) and the *mrtAB* mutant caused an equivalent rate of lethality during acute *Y. pseudotuberculosis* oral infection with 10^9^ bacteria (Fig. 7C).

### Multicopy expression of *mrtAB* results in enhanced resistance to ethidium bromide and increased sensitivity to pyocyanin

Only one report exists regarding the potential consequences of loss of MrtAB function. A phenotypic survey of *E. coli* genes revealed that a strain lacking the *E. coli mrtB* ortholog showed enhanced sensitivity to pyocyanin, an antimicrobial produced by *Pseudomonas aeruginosa* [28]. We did not detect a difference in the minimal inhibitory concentration (MIC) of pyocyanin resulting from the absence of MrtAB. We did observe, however, altered sensitivity under conditions predicted to overproduce MrtAB. The vector used in this study was a derivative of pACYC184, which is a multi-copy plasmid, and expression of *mrtAB* on this plasmid resulted in increased susceptibility to pyocyanin. The point mutation predicted to interfere with the ATPase activities of MrtB removed this pyocyanin sensitivity (Table 2).

**Table 2:** ATPase activity of MrtB is required for resistance to Ethidium Bromide.

	*Yptb*(P^−^) pGC1(empty)	*Yptb*(P^−^) Δ*mrtAB* pGC1(empty)	*Yptb*(P^−^) Δ*mrtAB* p*mrtA^+^B^+^*	*Yptb*(P^−^) p*mrtA^+^B^+^*	*Yptb*(P^−^) Δ*mrtAB* p*mrtA^+^B^+^flag*	*Yptb*(P^−^) Δ*mrtAB* pmrtA^+^B*-flag
EtBr	25	25	100	100	100	50
Acr Orange	50	50	50	50	ND	ND
Pyocyanin	1	1	0.25	0.25	0.25	2

Data are displayed as minimum inhibitory concentration (µg/mL), defined as: lowest concentration of toxic compound that resulted in less than half maximal growth in an overnight culture incubated without shaking, at 37°. + indicates wild type gene, while * indicates a gene with a point mutation in the Walker A box of the ATPase domain.

We next screened for altered sensitivity to compounds known to be substrates of efflux pumps. EtBr is a commonly utilized compound in the study of these pumps, as efflux provides the primary mechanism of EtBr resistance [29]. Multi-copy expression of *mrtAB* enhanced *Yptb* resistance to ethidium bromide (EtBr), increasing the minimal inhibitory concentration (MIC) by 4 fold (Table 2). The ability of MrtAB to confer enhanced resistance to EtBr strongly supports the hypothesis that MrtAB functions as an efflux pump. Providing additional support for this model, the ATPase function of MrtB was required for full EtBr resistance (Table 2). As the site of action of EtBr is in the bacterial cytoplasm, and that of pyocyanin may be in the periplasm [30], the phenotype of increased resistance to EtBr and enhanced susceptibility to pyocyanin is consistent with MrtAB exporting substrates across the inner membrane into the periplasmic space.

## Discussion

The importance of the virulence plasmid for the growth and spread of *Yptb* in various organs has been well documented using a variety of inoculation routes [4], [5]. Surprisingly, the absence of the plasmid appears to have an inconsequential effect on growth in the MLN in spite of the large number of known virulence factors encoded by the plasmid [5]. In this study, we have shown that a plasmid-deficient *Yptb* grew over 20X in both the liver and spleen and persisted for over a week at high levels in these organ sites (Fig. 1). This extraordinary ability to persist in the face of an antagonistic immune system implies that there is a range of unidentified *Yptb* chromosomal virulence factors. In this study we screened over 20,000 transposon insertion mutants for the ability to grow and persist for days in vivo, and identified a number of putative chromosomal virulence factors (Fig. 2, Table 1). This screen represents a larger number of *Yersinia* mutants screened than in all published *in vivo* genetic screens performed to date with *Yersinia pseudotuberculosis*, *Yersinia pestis*, and *Yersinia enterocolitica*, combined [10], [11], [12], [13]].

The 5 known virulence factors [10], [17], [21] that were hit in the screen served as validation of both the strategy that we used as well as the use of *Yptb*(P^−^) as the genetic background for the screen. While we anticipate that some of the *Yptb* genes identified in this study will only be required in the P^−^ background, we present the 5 hits in known virulence factors as evidence that many of the genes in Table 1 will also be required in the wild type background. In particular, most of the 11 genes that encode proteins involved in amino acid or purine synthesis are likely to be required in the wild type strain harboring the virulence plasmid. For example, *aroA* was shown to be essential for growth of *Yersinia enterocolitica* in mice [31], and both *aroA* and purine synthesis genes are essential for *Salmonella typhimurium* pathogenesis [22]. Mutants defective in amino acid and purine synthesis have been used to generate candidate vaccine strains for a variety of bacterial pathogens, and the genes identified in this study could provide additional platforms to use for vaccine development [22], [31], [32], [33].

One of the main goals of this study was to identify novel *Yptb* chromosomal virulence factors. Table 1 describes 18 candidate chromosomal virulence factors, none of which have been investigated in *Yptb*, and many of which have not been investigated in any pathogen. One of the few characterized virulence factors in this list is *apaH*, which is required for both invasion and adherence of *Salmonella enterica* to mammalian cells [34]. Two other well characterized genes are flagellar regulon members *flgD* and *flgC*, but it is unclear why these genes, which are essential for hook assembly, would play a role in this animal model when none of the other insertions in flagellar genes had any effect (Tables S1, S2, S3) [35]. In *Mycobacterium tuberculosis*, OppD was recently shown to reduce both apoptosis and inflammatory cytokine release from macrophages, which could have obvious parallels for *Yptb* trying to evade immune detection [36]. RodZ, a structural protein required for maintaining normal bacterial morphology, was also recently characterized as a regulator of post-transcriptional processing in *Shigella sonnei* [37].

Particularly interesting among the hits in the screen are the 14 genes predicted to be involved in LPS modification (Table 1). Two of the most severe defects seen in the screen correspond to genes encoding essential steps of the O-Ag synthesis pathway: *wecA* (YPK_4033), predicted to be involved in initiating synthesis of the O subunit, and *waaL* (YPK_3646), predicted to encode the ligase that attaches O-Ag to the lipid A core outer saccharide [38]. Interestingly, mutations in either of these genes also made *Yptb* unfit to grow at elevated temperatures (Table 1). Interpreting the phenotypes of mutations in the main O-Ag synthesis operon, YPK_3192 – YPK_3177, is difficult based solely on our screen, because any of these transposon insertions could disrupt expression of neighboring genes in the operon. This operon encodes proteins that produce the NDP-sugar subunits of O-antigen, as well as the O-Ag polymerase, flippase, and chain length regulator [39], [40]. The primary message from mutations in this operon is that some products are required for growth 37°C while, in general, most are required for growth of *Yptb*(P^−^) in deep tissue sites. YPK_3177, the predicted O-Antigen chain length regulator (*wzz*), on the other hand, is not essential for growth in mouse infection model we have used, as transposon insertions in this gene located at the end of the operon had no effect on growth in the liver (Table S1). RfaH, YPK_3937, is included in the O-Ag group because it is a bacterial elongation factor that is required for the expression of the O-Ag operon, among other genes.

A number of the same members of the homologous O-Ag synthesis operon were identified during a screen for *Y. enterocolitica* virulence factors [11], indicating that O-Ag production is also necessary in the presence of the virulence plasmid. Clearly, O-Ag plays a pivotal role in the pathogenesis of *Y. pseudotuberculosis*, and other Gram negative bacterial pathogens. Detailed studies of O-Ag status of *Y. enterocolitica* have shown that O-Ag production is critical for virulence, perhaps due to its role in the expression of other virulence factors, such as invasin and Ail [41]. Other studies have implicated the O-Ag of *S. enterica* in resistance to bile salts and anti-microbial peptides [42]. YPK_1834–1835 are part of an operon predicted to play a role in adding amino sugars to lipid A, which has also been implicated in resistance to anti-microbial peptides [43].

We decided to focus on *mrtAB*, as insertions in these genes resulted in two of the most significant growth deficits observed in the screen. *mrtAB* encodes for a poorly characterized, hypothetical ABC- type transporter. The *mrtAB* (previously annotated as *mdlAB* for “multi-drug resistance like”) operon is highly conserved in most Enterobacteriaceae, with the predicted protein sequence similarity being 85% conserved for *mrtA* in *E. coli*, *Shigella flexneri*, *S. enterica*, and *Klebsiella pneuomoniae*. One study showed that high levels of expression of *mrtAB* homologs in *S. enterica* correlated with increased resistance to a fluoroquinolone antibiotic, although deletion of these genes had no effect on fluoroquinolone resistance [44]. Another study examined the effect of *mrtAB* expression on resistance of *E. coli* to a variety of toxic compounds, and saw no effect [45].

In-frame deletions of either *mrtAB* or of the individual genes faithfully recapitulated the phenotypes from the screen, without any noticeable effect on growth *in vitro* (Fig. 2–3). Complementation *in trans* rescued the *mrtAB* deletion mutant to allow bacterial yields in the liver and spleen to near the levels of *Yptb* (P^−^) (Fig. 2). To our surprise, the putative transporter was entirely dispensable for growth of the fully virulent *Yptb* (P^+^) in these same organs as well as the small intestine and Peyer's patches (Fig. 4). Further examination revealed that, in the P^+^ background, *mrtAB* was only required in the mesenteric lymph nodes (Fig. 4).

A number of studies have demonstrated that productive infection by *Yptb* requires the same set of virulence factors in a variety of organ sites, such as the Peyer's patches, spleen, liver and lung [4], [5], [8]. The *Yptb* infection of the MLN is the anomaly, in that it is the only organ in which the virulence plasmid is not required [5]. That *mrtAB* is essential for infection of MLN provides additional evidence for the unique nature of the MLN interaction with *Yptb*. This raises the possibility that fully virulent *Yptb* persists in an entirely different selective environment in the MLN than in other organ sites. Since it is known that *Yptb* interacts with and preferentially translocates Yops into neutrophils *in vivo*, we first tested if there were altered neutrophil co-localization with bacteria in the spleen relative to MLN. In almost all bacterial foci in the MLN or the spleen, neutrophils similarly surrounded the bacterial microcolonies (Fig. 6). In addition, the observations that the virulence plasmid is capable of rescuing an *mrtAB* mutant in every organ except the MLN (Fig. 3–4), and that the virulence plasmid is dispensable for growth only in the MLN [5], led us to hypothesize that the plasmid-encoded type III secretion system substrates are not expressed by *Yptb* in the MLN. However, we were unable to detect any difference in expression of YopE using a YopE-mCherry reporter strain (Fig. 6A–B).

Characterization of the Wt *Yptb*(P+) infection of the spleen and MLN did not reveal any obvious differences that could explain the differential requirement for MrtAB in the colonization of these two organs (Fig. 4, 6). Therefore, to further interrogate the role of MrtAB in the MLN, we extended the oral infections to 4 days post-infection. Interestingly, the difference in bacterial burden in the MLN after 4 days of infection is largely erased, with the *mrtAB* mutant displaying only a 5 fold lower colonization of the MLN, as compared to an approximately 100 fold lower burden at 1 day post-infection (Fig. 4, 7). These results suggest that MrtAB is specifically required for initial MLN colonization, but does not play a role in post-colonization growth in this organ. Perhaps with a much lower dose of infection, the *mrtAB* mutant would be completely deficient for MLN colonization throughout the infection.

We hypothesized that the transport activity of MrtAB was required to support *Yptb* survival in mouse tissue sites. To test this, the Walker A box of MrtB was mutated, and tested for the ability to rescue the growth of *Yptb* (P^−^) Δ*mrtAB* in the spleen (Fig. 6). Mutation of the MrtB Walker A box strongly reduced the growth of *Yptb* mouse spleens, without noticeably altering the expression of the protein during growth in broth culture (Fig. 5A–B). This result indicates that the ATPase transport activity of the MrtAB ABC transporter is necessary for its role in promoting *Yptb* growth *in vivo*. The sequence and genetic organization of *mrtAB* is consistent with MrtAB forming a heterodimeric ABC family exporter. There exists a conserved TEVGERV motif in both MrtA and MrtB that is only found in ABC export systems [23], [46]. Furthermore, overexpression of MrtAB enhanced resistance to ethidium bromide, an activity that was largely dependent on the transport activity of MrtB (Table 2). Conversely, multicopy expression of *mrtAB* resulted in increased susceptibility to pyocyanin, a phenotype that required the MrtB ATPase. While the mechanism of pyocyanin toxicity is unclear [47], [48], [49], it is consistently reported to disrupt the cell membrane respiratory chain. In addition, pyocyanin was shown to block transport that is dependent on the proton motive force, consistent with a disruption of respiration [30]. Many of the components of the electron transport chain are accessible to or located within the periplasm. Therefore, expression of a transporter that moves pyocyanin into the periplasm, as a result of export across the inner membrane, could readily increase susceptibility to this toxic compound.

There are numerous potential roles that a bacterial transporter could play in virulence, including uptake of nutrients, resistance to toxic compounds, or secretion of an immunomodulatory bacterial compound. This study outlines several reasons to predict that MrtAB is involved in secretion, from homology to other ABC family exporters, to a function for MrtAB in providing resistance to a toxic compound. If MrtAB is involved in secretion of a toxic host compound, it is unlikely to be a toxic compound encountered in the small intestine, liver, spleen, or PP, as the Δ*mrtAB* mutant colonizes these organs at equal levels to Wt *Yptb* (Fig. 4B). It is possible that MrtAB is required for resistance to an unknown toxic host compound that is unique to the MLN; however, we consider this to be unlikely because MrtAB-deficient *Yptb* are eventually capable of colonizing the MLN at a level that is only moderately below that of Wt (Fig. 7A), and MrtAB is required for *Yptb*(P−) to survive in the liver and spleen (Fig. 3).

To determine if MrtAB was generally required for dissemination of *Yptb*(P+) from the intestine, we also tested the ability of the *mrtAB* mutant to colonize the spleen following oral infection. Interestingly, the *mrtAB* mutant was capable of colonizing the spleen at a level equal to Wt *Yptb*(P+), indicating that MrtAB is specifically required for transit to the MLN (Fig. 4B, 7B). While it is unknown how *Yptb* traffics to different organs during oral infection, it is clear that the MLN and the spleen are colonized independently, with the spleen being successfully colonized later during infection, following bacterial replication in the intestine, while the MLN is colonized within hours of infection [5], [19]. It is unknown how *Yptb* traffics to the MLN, though dendritic cells are thought to be important for *Salmonella enterica* serovar Typhimurium to gain access to this immune organ [50]. Perhaps MrtAB is required to survive interaction with trafficking dendritic cells, either by exporting an immunomodulatory bacterial compound, or providing resistance to a toxic dendritic cell compound. We speculate that during transit with an innate immune cell to the MLN, *Yptb* refrains from using the TTSS in order to avoid disrupting the normal trafficking of the host cell. This could explain why the virulence plasmid rescued an *mrtAB* mutant in all aspects of virulence except colonization of the MLN.

All pathogenic *Yersinia* species share a tropism for growth in lymph nodes, and lymph node pathology is commonly observed in infections with all *Yersinia* species, ranging from inflammation and swelling of regional lymph nodes (*Y. pestis* bubonic plague), to inflammation of the mesenteric lymph nodes (*Y. enterocolitica*, *Y. pseudotuberculosis* oral infections) [2]. Based on the high degree of conservation of MrtA and MrtB (99% ID in *Y. pestis*, 91–93% ID in *Y. enterocolitica*), we predict that MrtAB will play a role in the colonization of lymph nodes by all pathogenic *Yersinia* species, including *Y. pestis* and *Y. enterocolitica*. It will also be important to test the role of MrtAB in strains of *Y. pseudotuberculosis* that do not share the *phoP* mutation present in the YPIII strain used in this study [51]. Finally, MrtA and MrtB are also highly conserved in other bacterial pathogens that colonize the MLN, including *Salmonella enterica* serovar Typhimurium (76–79% ID), suggesting that transport mediated by MrtAB may be a common mechanism by which bacterial pathogens colonize this immune organ.

In conclusion, this study identified a number of candidate virulence factors in *Y. pseudotuberculosis*. MrtAB is the first mesenteric lymph node specific virulence factor identified in *Yersinia* species. Further study of this ABC transporter and its substrate(s) should provide valuable insight into the interaction of *Y. pseudotuberculosis* with the mesenteric lymph node and its unique requirements for establishing bacterial replication in this site.

## Materials and Methods

### Bacterial strains and genetics

All *Yersinia pseudotuberculosis* (*Yptb*) strains used in this study were derived from YPIII [5]. Plasmid deficient *Yptb* has been previously described [5]. In frame deletions were generated using pCVD442 and 500–800 bps upstream and downstream of the DNA to be removed, as described [5]. Primer sequences used to generate the *mrtAB* knockout construct were the following: *mrtAB* FOR1: attaGCATGCTTGCTGGAAACGTTTAAAGCGTTTGG, *mrtAB* REV1: attaGAATTCTAATTGTGCAAACAATCTCACGCAGTTT, *mrtAB* FOR2: attaGAATTC *AGGAGGTCGAAGC CGATGAAT*AAC, *mrtAB* REV2: attaGAGCTCTTGAAA TCAGCGCCATCCGCCAAT. For HA tagging of *mrtA* (YPK_3222), the HA sequence was inserted directly downstream of the ATG start codon of the operon. For the FLAG tagging of *mrtB* (YPK_3221), the FLAG sequence was inserted just upstream of the stop codon. The coding regions of the two genes are overlapping, which is why we avoided making any tags in the C terminus of MrtA or the N terminus of MrtB. *Yptb* were tagged with GFP by driving expression of GFP off the constitutive Tet promoter on pACYC184. The tetA::GFP promoter-gene fusion from pDW5 [52] was PCR-amplified with *Sph*I end sites and moved it into pACYC184 cut with *Sph*I. Forward primer: 5′ gatcgcatgcgaattctcatgtttgacagcttat 3′ Reverse primer: 5′ gccgccgcaaggaatggtgcatgc. This plasmid is very stable in vivo. For the construction of the *mrtAB* complementation plasmid (p*mrtAB*), pACYC184 was digested with *Eco*RV and *Sal*I, and the *mrtAB* operon was PCR-amplified with *Eco*RV and *Sal*I end sites. The entire intergenic sequence in between YPK_3223 and YPK_3222 (101 bps) was included upstream of the *mrtA* start codon, and the *mrtB* terminator was included after the gene. The primers used for the complementation vector were: CompFor: attaTCTAGAATAATTCACTAAAAAATCTGTTTATCAATGGT, and CompRev: attaGTCGACAAGTGA GTGAGTGAGTGAGTGAGT. A YopE reporter strain was constructed with a FLAG-mCherry sequence immediately following the *yopE* stop codon. An isogenic, unmarked T3SS reporter strain was constructed that contains FLAG-mCherry sequence immediately after the *yopE* stop codon (see Fig. S1, panel A). A DNA fragment containing the FLAG-mCherry sequence, flanked by ∼1 kb of genomic sequence on each side of *yopE* stop codon was constructed by PCR and cloned into the SacI and BamHI sites of pSR47s. The resulting plasmid (pSR47s-*yopE*-FLAG-mCherry) was introduced into *E. coli* DH5α λpir and integrated onto the *Y. pseudotuberculosis* virulence plasmid via triparental mating using the helper strain HB101(RK600).

### Media and growth conditions

All *Yptb* cultures were grown in 2XYT, except for determination of the MIC (below). Kanamycin (30 µg/mL) for selection of transposon and Irgasan (2 µg/mL) for selection for *Yptb* were used in the production of *Yptb* transposon mutant libraries. Chloramphenicol (25 µg/mL )was used in selection for pACYC184 derived complementation plasmids. For IV, oral, or IP infections, *Yptb* cultures were grown at 26°C overnight, rolling, prior to infection. For *in vitro* growth for measuring MrtB protein levels by Western blot, bacteria were grown overnight in LB, and backdiluted 1∶40 the following morning in LB. Cultures were allowed to grow for 90 minutes at 26°C, then half the samples were switched to 37°C and half were left at 26°C, and the cultures were growth for an additional 90 minutes prior to protein isolation. Mouse anti-FLAG was used as a primary antibody, overnight at 4°C, and Cy5 Goat anti-Mouse was used as a secondary antibody. Westerns were visualized on a Fuji FLA-9000.

### Generation of mariner transposon mutant libraries in *Yptb* (P^−^)

The vector pSC189 containing Himar1 [53] was mutated on one end of the transposon recognition sequence to produce an *Mme*I restriction site, as described [20]. To perform transpositions in a *Yptb* strain, the Himar1(*Mme*I) transposon was introduced into YPIII(P^−^) by mating with SM10λ*pir*. Briefly, 25 mL of YPIII(P^−^) was grown overnight (O/N) in 2XYT broth at 26°C, and 75 mL of SM10λ*pir*(pSC189Himar1(*Mme*I) was grown O/N at 37°C in LB containing 30 µg/mL Kan and 100 µg/mL Amp. The SM10λpir cultures were washed 3X with PBS, pelleted, and resuspended in the YPIII(P^−^) culture. Mating was allowed to proceed for 16–24 hours at 37°C in the spent *Yptb* culture, standing. Bacteria were then pelleted, resuspended in 5 ml 2XYT, and spread on 10 LB plates containing 30 ug/ml kanamycin and 2 µg/ml irgasin. Libraries of approximately 10,000 colonies were scraped off plates, pelleted, resuspended in 50% glycerol and stored at −80°C.

### Genetic screens

Libraries of 10,000 Himar1 mutants were adjusted to yield a total of 200,000 colonies on LB medium, scraped and resuspended in 2XYT, and small aliquots were used to start O/N cultures in 2XYT at 26°C. Tail vein injections of 1×10^5^ bacteria were introduced into mice, organs were isolated and homogenized at various times post infection, and bacteria were isolated by plating for all bacteria, on LB Medium containing 30 ug/ml kanamycin and 1 ug/ml irgasan. Colonies were scraped off plates and genomic DNA was isolated using Qiagen DNeasy kit. Samples were prepared for Illumina sequencing, as described [20], [54]. For 26°C vs. 37°C screen, both 10,000 Himar1 mutant libraries were plated out and combined. O/N cultures were grown at 26°C, diluted into 2XYT the following day, and grown O/N at either 26°C or 37°C. The temperature selection screen was thus a screen for both growth and stationary phase at 37°C.

#### Screen data analysis

Each overnight culture of an individual library of 10,000 transposon mutants was plated out for over 200,000 colonies, which were used to generate genomic DNA (above). This is defined as an Input sample. For each library, there were at least 2 Input samples, with a corresponding set of Output samples, defined as the colonies from an individual infected liver derived from a given Input injection dose. After Illumina sequencing, the number of reads for each gene in the Output sample was normalized for amount of DNA added to sequencing run (total number of reads) and normalized for the number of unique insertions in a particular pool. This provides a value for each gene, for the relative abundance of clones containing a transposon insertion in gene X within the pool. Insertions in the first 5% or last 10% of a gene were discarded and all remaining values for insertions within a single gene were summed. Finally, the values for each gene in the Output liver samples were divided by the values in the corresponding Input sample. This provides a ratio of the relative abundance of clones containing a transposon insertion in Gene X in the Output liver sample, divided by the relative abundance of clones containing a transposon insertion in Gene X in the Input sample. The Log_2_ value of this ratio was used for further statistical analysis, including determining the average ratio and standard deviation (s.d.).

### Mouse infections and histology

All mice used were 8–10 weeks old. All intravenous (IV) infections that were analyzed by CFU were performed in C57BL/6 mice. For *Yptb* (P^−^), mice were infected IV with 1×10^5^ bacteria. For WT, mice were infected IV with 1×10^3^ bacteria. All oral infections were performed in BALB/c mice for ease of Peyer's patch isolation. Intraperitoneal infections were also performed in BALB/c mice. For oral infections, food was removed from cages 16 hours before oral inoculation with 2×10^9^ *Yptb*. For Peyer's Patch quantification of CFU, all visible PP from a single animal were combined prior to homogenization and plating. For small intestine CFU quantification, the 5 cm of small intestine upstream of the caecum were removed. For both PP and small intestine, homogenates were plated on LB with 1 µg/mL Irgasan. For histology, inoculations with GFP tagged bacteria were performed as all other inoculations, and organs were fixed in 4% paraformaldehyde for 3 hours, then flash frozen in Sub Xero freezing media (Mercedes Medical). 10 µm sections were cut using a cryostat microtome, and stained with Hoechst (1∶10,000). For neutrophil staining, monoclonal anti-Ly6G clone 1A8 (BD Pharmingen) was used at 1∶100. For quantification of the YopE reporter strain, tissue sections were prepared as described above, and imaged using a Nikon A1R confocal. Images were quantified using ImageJ, with each microcolony analyzed for median mCherry fluorescence, normalized to median GFP, which was constitutively expressed.

### Ethics statement

This study was carried out in strict accordance with the recommendations in the Guide for the Care and Use of Laboratory Animals of the National Institutes of Health. The protocol was approved by the Tufts University Institutional Animal Care and Use Committee (IACUC). Our approved protocol number is B2010-100. All efforts were made to minimize suffering: animals were carefully monitored following infection and were euthanized prior to or directly upon exhibiting substantial signs of morbidity. Animals were euthanized by C0_2_ asphyxiation followed by cervical dislocation.

### MIC analysis

*Yptb* cultures were grown in 2XYT overnight at 26°C, with chloramphenicol 25 µg/mL if they contained a pACYC184 derivative. Bacteria were diluted in LB broth, and used to inoculate 96 well plates containing 2 fold serial dilutions of Acridine Orange, Ethidium Bromide, or Pyocyanin, with chloramphenicol 25 µg/mL if they contained a pACYC184 derivative. Bacteria were grown overnight at 37°C without shaking, and OD600 was measured 18 hours later. The MIC is defined as the lowest concentration of toxic compound that results in half maximal growth (i.e. half the A_600_ of the untreated control), and the values presented in Table 2 are the average of 6 replicates.

## Supporting Information

Figure S1 **Construction and characterization of YopE reporter strain.** **A**) *yopE* reporter strain (*yopE*-STOP::FLAG-mCherry) construction. FLAG-mCherry sequence was inserted immediately after the yopE stop codon to serve as a reporter for *yopE* expression. **B**) *yopE* reporter expression is properly regulated. Bacteria were grown at 37°C (Wt and Δ*lcrF* containing *yopE*-STOP::FLAG-mCherry) or 26°C (Wt containing *yopE*-STOP::FLAG-mCherry) and bacteria were visualized by Phase contrast and fluorescence microscopy. **C**) Reporter expression does not affect endogenous yopE expression. Wild-type, two Wt *yopE*-STOP::FLAG-mCherry isolates, and an Δ*lcrF yopE*-STOP::FLAG-mCherry isolate were grown in yop inducing conditions. Bacteria were lysed and proteins analyzed by Western blotting. **D–E**) Reporter strain fluorescence decreases in the absence of yop expression. Bacteria were grown in yop-inducing conditions (37°C, low Ca2+), washed, and shifted to non-inducing conditions (26°C, high Ca2+). Samples were taken every two hours and imaged by phase and fluorescence microscopy (**D**). Fluorescence was quantified on a per-bacterium basis (**E**).
(PDF)

Table S1 **Screen data for genes hit in both libraries.** **Column C–F:** The number of reads for each gene in the Input samples normalized for the amount of DNA added to sequencing run (total number of reads) and normalized for the number of unique insertions in a particular pool. **Column G–AC:** All the Output Liver samples, normalized as in C–F, then divided by the values in the corresponding Input sample. **Column AD–AM:** Statistical analysis and annotation, including the average ratio of Output/Input, the Log_2_ value of this ratio, the number of Standard Deviations away from the mean, and a reference to the 26° vs 37° growth (Table S4).
(XLS)

Table S2 **Screen data from genes only hit in Library A.** Similar analysis as in Table S1, but only including data from genes hit Library A and not hit in Library B. **Column B, H:** The number of reads for each gene in Library A Input sample normalized for the amount of DNA added to sequencing run (total number of reads) and normalized for the number of unique insertions in a particular pool. **Column C–G, I–M:** Output Liver samples, normalized as in B and H, then divided by the values in the corresponding Input sample. **Column O–U:** Statistical analysis, including the average ratio of Output/Input, the Log_2_ value of this ratio, and the number of Standard Deviations away from the mean.
(XLS)

Table S3 **Screen data from genes only hit in Library B.** Similar analysis as in Table S1, but only including data from genes hit Library B and not hit in Library A. **Column B, H:** The number of reads for each gene in Library B Input sample normalized for the amount of DNA added to sequencing run (total number of reads) and normalized for the number of unique insertions in a particular pool. **Column C–G, I–P:** Output Liver samples, normalized as in B and H, then divided by the values in the corresponding Input sample. **Column Q–Y:** Statistical analysis, including the average ratio of Output/Input, the Log_2_ value of this ratio, and the number of Standard Deviations away from the mean.
(XLS)

Table S4 **26° vs. 37° growth in vitro.** Libraries A and B were combined and grown overnight at 26°, diluted into 2XYT the following day, and grown overnight at either 26°C or 37°C. **Column B, C:** The number of reads for each gene from samples grown at 26°C, normalized for the amount of DNA added to sequencing run (total number of reads) and normalized for the number of unique insertions in a particular pool. **Column D, E:** Similar analysis to Column B and C, only from samples grown at 37°C. **Column F–N:** Statistical analysis, including the ratio of 26°C vs. 37°C values, the Log_2_ value of this ratio, and the number of Standard Deviations away from the mean.
(XLSX)
