# Supplementary figures and images for: Identification of MrtAB, an ABC Transporter Specifically Required for Yersinia pseudotuberculosis to Colonize the Mesenteric Lymph Nodes
Source: PLoS Pathog. 2012 Aug 2;8(8):e1002828. doi: 10.1371/journal.ppat.1002828 (PMC3410872; doi:10.1371/journal.ppat.1002828)

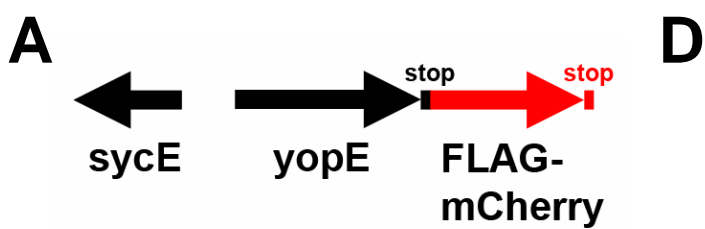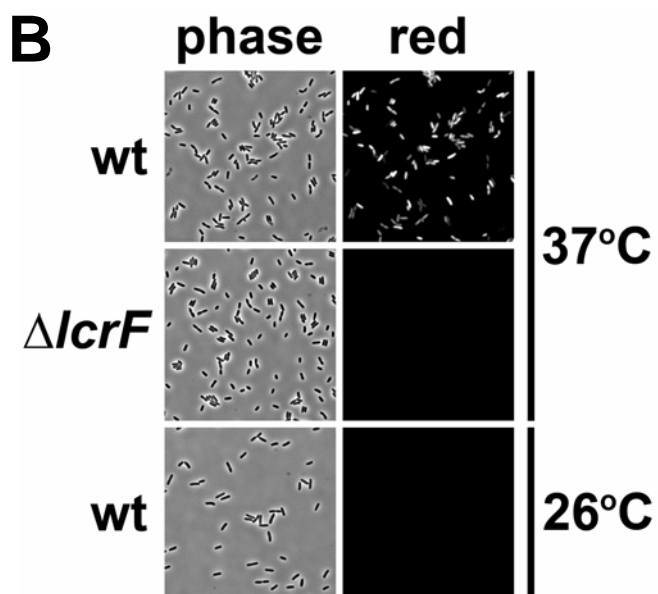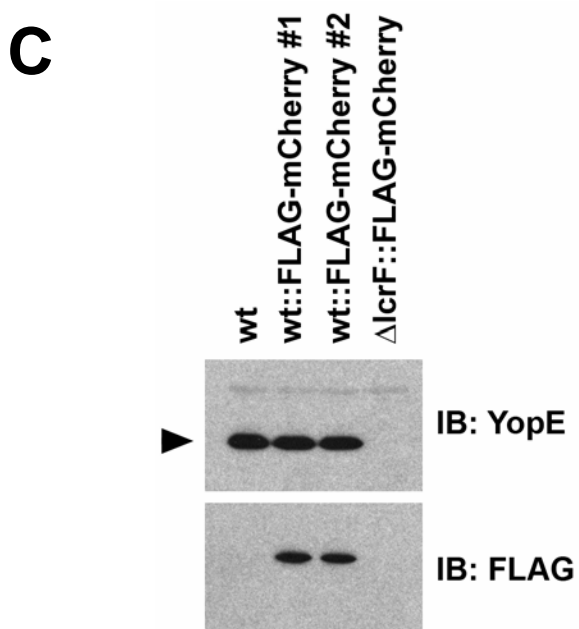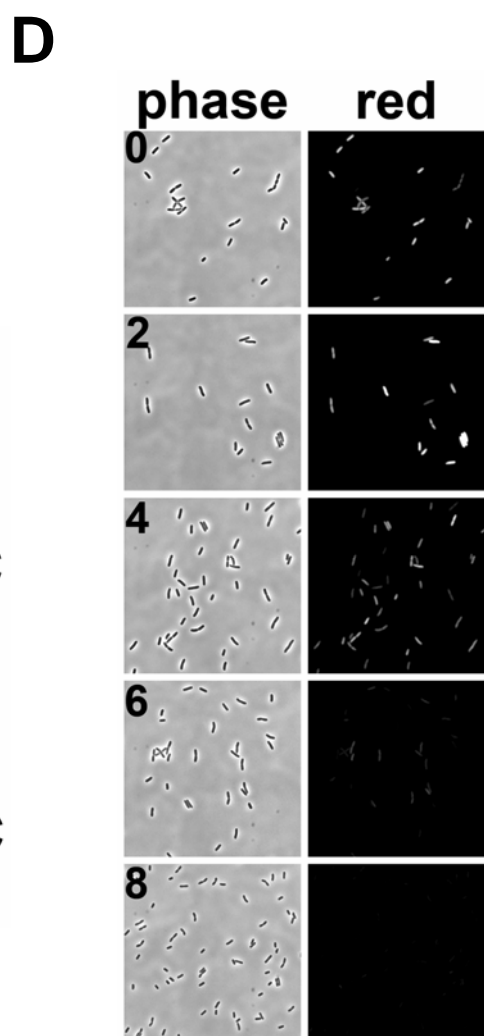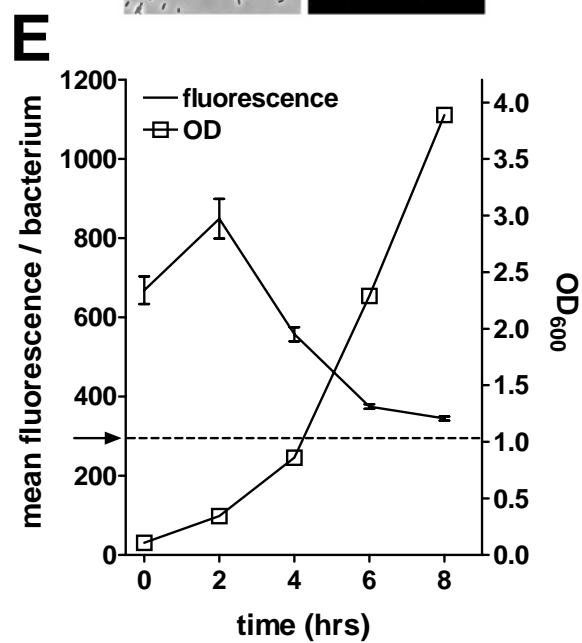

Supplement: Figure S1 — Construction and characterization of YopE reporter strain. A) yopE reporter strain (yopE-STOP::FLAG-mCherry) construction. FLAG-mCherry sequence was inserted immediately after the yopE stop codon to serve as a reporter for yopE expression. B) yopE reporter expression is properly regulated. Bacteria were grown at 37°C (Wt and ΔlcrF containing yopE-STOP::FLAG-mCherry) or 26°C (Wt containing yopE-STOP::FLAG-mCherry) and bacteria were visualized by Phase contrast and fluorescence microscopy. C) Reporter expression does not affect endogenous yopE expression. Wild-type, two Wt yopE-STOP::FLAG-mCherry isolates, and an ΔlcrF yopE-STOP::FLAG-mCherry isolate were grown in yop inducing conditions. Bacteria were lysed and proteins analyzed by Western blotting. D–E) Reporter strain fluorescence decreases in the absence of yop expression. Bacteria were grown in yop-inducing conditions (37°C, low Ca2+), washed, and shifted to non-inducing conditions (26°C, high Ca2+). Samples were taken every two hours and imaged by phase and fluorescence microscopy (D). Fluorescence was quantified on a per-bacterium basis (E). (PDF) [file ppat.1002828.s001.pdf]
